# Development and validation a radiomics nomogram for predicting thymidylate synthase status in hepatocellular carcinoma based on Gd-DTPA contrast enhanced MRI

**DOI:** 10.1186/s12885-023-11096-7

**Published:** 2023-10-17

**Authors:** Zongren Ding, Yijun Wu, Guoxu Fang, Zhaowang Lin, Kongying Lin, Jun Fu, Qizhen Huang, Yanyan Tang, Wuyi You, Jingfeng Liu, Yongyi Zeng

**Affiliations:** 1 Department of Hepatopancreatobiliary Surgery, Mengchao Hepatobiliary Hospital of Fujian Medical University, Xihong Road 312, Fuzhou, 350025 China; 2 Department of Radiology, Mengchao Hepatobiliary Hospital of Fujian Medical University, Fuzhou, 350025 China; 3 Department of Radiotherapy, Mengchao Hepatobiliary Hospital of Fujian Medical University, Fuzhou, 350025 China; 4 Fujian Provincial Cancer Hospital, Fuzhou, 350025 China

**Keywords:** Radiomics, Thymidylate synthase, Hepatocellular carcinoma, Nomogram

## Abstract

**Objectives:**

The purpose of this study was to develop and validate a radiomics nomogram for predicting thymidylate synthase (TYMS) status in hepatocellular carcinoma (HCC) by using Gd-DTPA contrast enhanced MRI.

**Methods:**

We retrospectively enrolled 147 consecutive patients with surgically confirmed HCC and randomly allocated to training and validation set (7:3). The TYMS status was immunohistochemical determined and classified into low TYMS (positive cells ≤ 25%) and high TYMS (positive cells > 25%) groups. Radiomics features were extracted from the arterial phases and portal venous phase of Gd-DTPA contrast enhanced MRI. Least absolute shrinkage and selection operator (LASSO) were applied for generating the Rad score. Clinical data and MRI findings were assessed to build a clinical model. Rad score combined with clinical features was used to construct radiomics nomogram.

**Results:**

A total of 2260 features were extracted and reduced to 7 features as the most important discriminators to build the Rad score. InAFP was identified as the only independent clinical factors for TYMS status. The radiomics nomogram showed good discrimination in training (AUC, 0.759; 95% CI 0.665–0.838) and validation set (AUC, 0.739; 95% CI 0.585–0.860), and showed better discrimination capability (P < 0.05) compared with clinical model in training (AUC, 0.656; 95% CI 0.555–0.746) and validation set (AUC, 0.622; 95% CI 0.463–0.764).

**Conclusions:**

The radiomics nomogram shows favorable predictive efficacy for TYMS status in HCC, which might be helpful for the personalized treatment of HCC.

**Strengths and limitations of this study**.

1. Fluorouracil is one of the most commonly used chemotherapy drugs for hepatocellular carcinoma.
2. Thymidylate synthase expression level were a key determinant for therapeutic responsiveness to fluorouracil.
3. Detecting of TYMS not only has guiding significance for the selection of chemotherapy drugs for HCC patients, but also has certain value for the judgment of prognosis.
4. The present study proved that the Gd-DTPA contrast-enhanced MR-based radiomics model has favorable predictive value for predicting TYMS status in HCC.

## Introduction

Hepatocellular carcinoma (HCC) is the most common primary liver cancer and the second most common cause of cancer death worldwide [1]. Because of the occult symptoms of HCC, most patients were intermediate or advanced and unresectable when diagnosed. For unresectable HCC, various systemic therapies are available, such as molecular targeted agents, immunotherapy or chemotherapy [1]. However, the application of chemotherapy in HCC is very limited because HCC has also been shown to be chemoresistant to the most common chemotherapies. Moreover, chemoresistance leads to the poor prognosis of HCC and is also a major cause of tumour recurrence and metastasis in HCC [2]. With the in-depth study of the mechanism of chemoresistance, many expression products of key drug resistance genes have been found, such as thymidylate synthase (TYMS), DNA topoisomerases-II (TopoII), P-gp and tubulin beta-III (βIII-tubulin) [3–6].

TYMS is a key rate-limiting enzyme in folate metabolism, catalysing the conversion of deoxyuridine monophosphate (dUMP) to deoxythymidine monophosphate (dTMP) [7]. This conversion essentially influences DNA repair, methylation and synthesis through the production of nucleotides. Several preclinical studies have shown that TYMS expression levels are a key determinant for therapeutic responsiveness to fluorouracil, which is one of the most commonly used chemotherapy drugs for HCC [8–10]. Therefore, it was reported that TYMS gene expression may be an independent predictor of survival in HCC [11].

Typically, TYMS status is assessed by pathological methods. HCC is mostly diagnosed at advanced stages of disease, and pathological evidence is not currently available. Biopsies are an invasive and unpleasant procedure and are unnecessary for these patients. Hence, there is an urgent need to develop a noninvasive and accurate method to predict TYMS status in HCC for the guidance of chemotherapies and the prediction of prognosis.

Radiomics is an emerging field involving the extraction of high-throughput data from quantitative imaging features and the subsequent combination of this information with clinical data in an attempt to provide prognostic and predictive information from imaging features alone. Many studies have demonstrated the ability of radiomics or radiogenomics to predict gene expression in HCC [12, 13]. To the best of our knowledge, there is no literature describing a radiomics signature that could facilitate the noninvasive detection of TYMS status in HCC. Therefore, this study aimed to develop and validate a radiomics nomogram for predicting TYMS status in HCC based on Gd-DTPA contrast-enhanced MRI.

## Materials and methods

### Patients

In this single-centre retrospective study, the medical records were viewed to collect consecutive patients from January 2019 to August 2020. The inclusion criteria were as follows: (a) HCC diagnosis with pathological evidence, including biopsies and surgery, (b) complete clinical and pathologic data, and (d) patients who underwent Gd-DTPA contrast-enhanced MRI less than 15 days before achieving pathological evidence. The exclusion criteria were (a) absence of high-quality Gd-DTPA contrast-enhanced MRI (no image artefact or an incomplete sequence that can affect the radiomics analysis) and (b) incomplete medical records such that required clinical data were not available. We developed and validated the model using the TRIPOD (transparent reporting of a multivariable prediction model for individual prognosis or diagnosis) guidelines [14].

This study was approved by the Institutional Ethics Committee of Mengchao Hepatobiliary Hospital of Fujian Medical University (2020_072_01), and written informed consent was obtained from all study participants. The studies were performed in accordance with the ethical standards as outlined in the 1964 Declaration of Helsinki and its later amendments or comparable ethical standards.

Patient and Public Involvement: The patients or the public WERE NOT involved in the design, conduct, reporting or dissemination plans of our research.

### Clinical data and TYMS status assessment

A standardized data form was created to collect all relevant clinical information. The form included 28 items that were categorized into the 4 following groups: (1) demographics and clinical characteristics of the patients (including age, gender, HbsAg, HBeAG, HBV-DNA, HCVAb and ascites); (2) laboratory variables including Child‒Pugh class, total bilirubin (TBIL), albumin (ALB), alanine aminotransferase (ALT), aspartate aminotransferase (AST), prothrombin time (PT), international normalized ratio (INR), alpha-fetoprotein (AFP), carcinoembryonic antigen (CEA), cancer antigen 125 (CA125), and carbohydrate antigen 19 − 9 (CA19-9); (3) liver and tumour characteristics, such as liver cirrhosis, tumour number and diameter (of the largest lesion in multinodular HCC), tumour margin, capsule and haemorrhage (targeted HCC), arterial hyperenhancement status (including early enhancement and washout) and venous invasion on MR scan; and (4) TYMS status.

The histological HCC evaluation was performed by two pathologists with > 10 years of experience who initially fixed the specimens in 10% formalin and embedded them in paraffin. The resulting 3–4 mm paraffin sections were prepared for immunohistochemical analysis. Thymidylate synthase (TYMS) expression was detected using a commercially available anti-human TYMS mouse monoclonal antibody (TS106, Maixin Biotechnology). We divided TYMS expression in HCC into high-expression (tumour cell positive rate > 25%) and low-expression (tumour cell positive rate ≤ 25%) cohorts.

### MR Image acquisition and image processing

The MRI examination was performed using a 3.0 T magnetic resonance scanner (Magnetom Verio, Siemens Healthcare, Erlangen, Germany), an 8-channel phased array body coil, and a high-pressure syringe. The contrast agent was Gd-DTPA (Gd - DTPA, BeiLu Pharmaceutical), the dosage was 0.2 ml/kg, the speed was 2.5 ml/s, and the contrast was flushed with 20 ml of normal saline. The preparation before the scan: patients were instructed to fast (no food or water) for at least four hours before scan, psychological guidance and breathing training (calm breath holding at the end of the breath) were conducted. Contrast-enhanced axial T1-weighted images (CE - T1) were acquired using a three-dimensional volumetric interpolated breath-hold examination (3D - VIBE) sequence (TR = 4.16 ms, TE = 2.01 ms, FOV = 380 × 308 mm, matrix = 320 × 320 × 75%, slice thickness = 3 mm, spacing = 3 mm, FA = 16°, and NEX = 1) with multiphase contrast. Arterial phase, portal venous phase and delayed phase images were acquired after contrast administration at 20–30, 60–70, 120–1180 s for each patient, with breath holding in all phases.

The lesions were segmented manually using 3D-Slicer (version 4.10.2), and the arterial phases and portal venous phase of T1 images were used to indicate the volume of interest (VOIs) by drawing the outline of tumour tissues layer-after-layer and avoiding bile duct and vessels by radiologist 1 and radiologist 2. If there were multiple lesions, only the largest lesions were segmented. PyRadiomics (version 2.1) implementation in 3D-Slicer was utilized for further preprocessing steps and radiomics feature extraction. We adopted resampling as a preprocessing method, which was performed to obtain a voxel size of 1 × 1 × 1 mm3 via trilinear interpolation before feature calculation [15]. A fixed bin width of 25 was used for image discretization. Image reconstruction was performed by applying wavelet decomposition filtering and Laplacian of Gaussian filtering with sigma values of 0.5, 1.0, and 1.5. Seven common feature groups were extracted from the filtered and original images in three dimensions, including first order, grey-level dependence matrix (GLDM), grey-level cooccurrence matrix (GLCM), grey-level run length matrix (GLRLM), grey-level size zone matrix (GLSZM), neighbouring grey tone difference matrix (NGTDM) and shape.

### Construction of the clinical model and radiomics model

The intraclass correlation coefficients (ICCs) are one of the reliability coefficients used to measure and evaluate inter-observer reliability and test-retest reliability. We use the ICCs to evaluate intra-observer and inter-observer reliability and reproducibility of feature extraction as previous radiomics articles describe [15, 16]. Thirty samples were randomly chosen and delineated by the two radiologists. Radiologist 1 delineated the VOIs on arterial phases and portal venous phase of T1 images twice within one week under the same standard to assess the intra-observer reproducibility, and radiologist 2 independently delineated the VOIs once to assess the inter-observer agreement by comparing the results with the radiomics features extracted from the VOIs delineated by the radiologist 1 [16]. Radiomics features were selected when the intraclass correlation coefficient (ICC) was > 0.8. Radiologist 1 finished the remaining samples.

Before radiomics feature selection, z score normalization was employed to eliminate different feature magnitudes by scaling values to a mean of 0 and a standard deviation of 1 [16]. Then, the samples were randomly grouped into training and validation sets at a ratio of 7:3. The training set was used for radiomics feature selection and construction of the models, and the validation set was used to evaluate the diagnostic performance of the models. Least absolute shrinkage and selection operator regression (LASSO) was employed for the selection of features, with penalty parameter tuning conducted by 10-fold cross-validation to compile a radiomics signature [17]. Then, a correlation analysis was carried out to exclude the features with high correlation. Multivariate logistic regression was applied to generate the Rad score.

Univariate regression analysis was applied to compare the differences in the clinical data and MRI findings between the two groups (low and high TYMS groups), and multiple logistic regression analysis was used to build the clinical model using the significant variables from the univariate analysis as inputs. Odds ratios (ORs) as estimates of the relative risk with 95% confidence intervals (CIs) were obtained for each risk factor. The Rad-score combined with clinical features in the clinical model was used to construct a radiomics nomogram.

### Statistics

Statistical analysis was performed using R (version 3.6.3). Categorical variables were compared using the χ2 test or Fisher’s exact test. Continuous variables were expressed as the median [Q1, Q3] and compared using Student’s t test or the Mann‒Whitney U test. Serum AFP levels were normalized with a natural logarithm transformation to reduce the effect of small differences. Variables that reached statistical significance in the univariate analysis were considered for the multivariate model based on multivariate binary logistic regression. LASSO was implemented using ‘glmnt’. The DeLong test was used to measure the differences in ROC curves [16]. Model fit was assessed by calibration plots via 1000 bootstrap resamples. The clinical utility of the models was evaluated by decision curve analysis. P < 0.05 was considered statistically significant.

## Results

### Patient characteristics

A total of 147 patients with HCC (127 men and 20 women; mean age, 54.9 ± 13.3 years) were enrolled in this study. The clinical data and MRI findings of HCCs in the training and validation sets are shown in Table 1. TYMS status was identified as high in 45 (43.7%) patients and 23 (52.3%) patients in the two sets, respectively (P = 0.438). All clinical data and MRI findings showed no significant difference between the two sets (P > 0.05).

**Table 1:** Characteristics of patients in the training and validation sets

Clinical factors	Training set (n = 103)	Validation set (n = 44)	P
**Age**, years	59.0 [47.0, 65.0]	55.0 [43.8, 65.0]	0.372
**Gender**, Female/ Male	14 (13.6%)/89 (86.4%)	6 (13.6%)/38 (86.4%)	1
**HbsAg**, positive/ negative	96 (93.2%)/7 (6.8%)	39 (88.6%)/5 (11.4%)	0.55
**HBeAG**, positive/ negative	17 (16.5%)/86 (83.5%)	12 (27.3%)/32 (72.7%)	0.202
**HBV-DNA**, copies/ml ≤ 10^4/>10^4	78 (75.7%)/25 (24.3%)	30 (68.2%)/14 (31.8%)	0.456
**HCVAb**, positive/ negative	4 (3.9%)/99 (96.1%)	0 (0%)/44 (100%)	0.44
**Child-Pugh class**, A/B	86 (83.5%)/17 (16.5%)	39 (88.6%)/5 (11.4%)	0.584
**TBIL**, mmol/l	14.5 [11.8, 19.8]	15.2 [12.7, 18.1]	0.391
**ALB**, g/l	38.0 [34.0, 41.0]	38.0 [36.0, 39.3]	0.242
**ALT**, m/l	43.0 [25.5, 68.0]	28.5 [22.5, 49.8]	0.231
**AST**, m/l	41.0 [28.0, 75.0]	36.5 [24.8, 50.3]	0.851
**PT**, s	13.1 [12.6, 13.6]	13.4 [12.9, 13.9]	0.372
**INR**	0.990 [0.945, 1.05]	1.02 [0.970, 1.06]	0.354
**InAFP**	4.49 [2.28, 6.15]	4.49 [1.60, 7.60]	0.535
**CEA**, ng/ml	2.60 [1.85, 4.05]	2.95 [1.60, 3.80]	0.327
**CA125**, U/ml	12.0 [8.86, 21.0]	12.0 [7.68, 19.8]	0.175
**CA19-9**, U/ml	16.4 [8.45, 28.9]	15.7 [8.86, 27.3]	0.776
**Liver cirrhosis**, Absent/ Present	55 (53.4%)/48 (46.6%)	23 (52.3%)/21 (47.7%)	1
**Ascites**, Absent/ Present	87 (84.5%)/16 (15.5%)	35 (79.5%)/9 (20.5%)	0.626
**Tumour number**, Solitary/ Multiple	81 (78.6%)/22 (21.4%)	31 (70.5%)/13 (29.5%)	0.392
**Tumour diameter**, cm	4.40 [2.40, 7.40]	5.25 [2.68, 7.10]	0.827
**Tumour margin**, Non smooth/ Smooth	69 (67.0%)/34 (33.0%)	32 (72.7%)/12 (27.3%)	0.622
**Tumour capsule**, Absent/ Present	56 (54.4%)/47 (45.6%)	26 (59.1%)/18 (40.9%)	0.729
**Hemorrhage**, Absent/ Present	93 (90.3%)/10 (9.7%)	39 (88.6%)/5 (11.4%)	0.995
**Venous invasion**, Absent/ Present	83 (80.6%)/20 (19.4%)	35 (79.5%)/9 (20.5%)	1
**Early enhancement**, Absent/ Present	22 (21.4%)/81 (78.6%)	7 (15.9%)/37 (84.1%)	0.593
**Washout**, Absent/ Present	30 (29.1%)/73 (70.9%)	8 (18.2%)/36 (81.8%)	0.237
**TYMS**, Low/ High	58 (56.3%)/45 (43.7%)	21 (47.7%)/23 (52.3%)	0.438

**Note**: HbsAg, Hepatitis B surface antigen; HBeAG, hepatitis B e antigen; HCVAb, Hepatitis C Virus Antibody; TBIL, total bilirubin; ALB, albumin; ALT, alanine transarninase; AST, Aspartate transaminase; PT, prothrombin time; INR, international normalized ratio; AFP, alpha-fetoprotein; CEA, carcinoembryonic antigen; CA125, cancer antigen 125; CA19-9, carbohydrate antigen 19 − 9; TYMS, thymidylate synthase

### Radiomics features selection

**Fig. 1:**
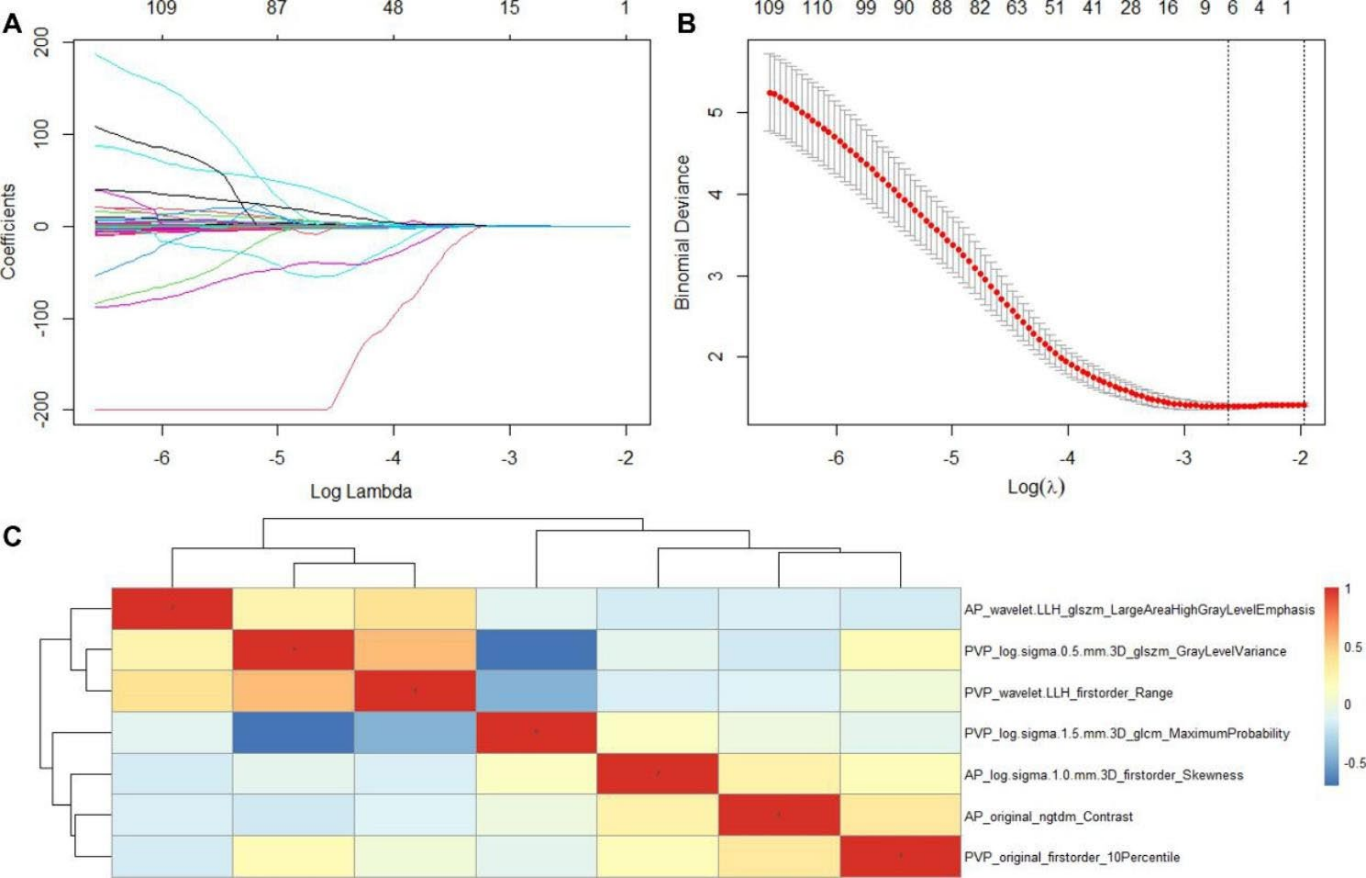
(**A**) LASSO regression analysis of 1529 features. (**B**) The AUC curve was plotted by tuning parameter (λ) selection performed by 10-fold cross-validation. Vertical lines on the left and right denote the minimum criterion and 1-standard error criterion (1 - SE), respectively. The minimum criterion was applied and 7 features was selected. (**C**) The correlation analysis heatmap of 7 features screened by LASSO.

We extracted 2,260 radiomics features in each patient from the arterial phase and portal venous phase of ceMRI. For intra- and interobserver agreement, 1529 radiomics features were selected with an intraclass correlation coefficient (ICC) > 0.8, and seven hundred thirty-one features were excluded. Then, 1529 features were subjected to LASSO regression, and seven features (three and four features, respectively, from arterial phase and portal venous) were selected with the best tuned regularization parameter Log (λ) of -2.62 under the minimum criterion found by 10-fold cross validation. A Pearson correlation analysis of the seven features showed that all features were weakly correlated, with a Pearson correlation coefficient less than 0.80 (Fig. 1). The Rad score was built according to the weight coefficient of the selected seven features in the LASSO regression. The Rad score showed statistically significant differences between low and high TYMS, and there was a significant difference in the median Rad score of low TYMS: -0.451 [− 0.621, − 0.259]; median Rad score of high TYMS: −0.157[− 0.426, 0.058] in the training set and median Rad score of low TYMS: -0.535 [− 0.690, -0.251]; median Rad score of high TYMS: −0.125 [− 0.355, 0.012] in the validation set.

### Construction of the clinical model and radiomics model

In the training set, the univariate analysis showed that PT, INR, and InAFP reached statistical significance (P < 0.05) when comparing the high and low TYMS groups (Table 2), while the multivariate analysis identified InAFP (OR 1.291; 95% CI 1.077–1.567; p = 0.007) as the only independent predictor of TYMS, and InAFP was used to build the clinical model. Moreover, PT (OR = 0.979, 95% CI 0.003–10.141, P = 0.985) and INR (OR = 0.026, 95% CI 6.659e-13–2.069e + 08, P = 0.756) were excluded. The Rad score combined with InAFP was used to construct the radiomics nomograms.

**Table 2:** Univariate analysis for clinical data and MRI findings associated with the TYMS status in the training set

Clinical factors	Low TYMS (n = 58)	High TYMS (n = 45)	P value
**Age**, years	61.0 [51.3, 66.0]	54.0 [41.0, 64.0]	0.119
**Gender**, Female/ Male	10 (17.2%)/48 (82.8%)	4 (8.9%)/4 (8.9%)	0.349
**HbsAg**, positive/ negative	53 (91.4%)/5 (8.6%)	43 (95.6%)/2 (4.4%)	0.659
**HBeAG**, positive/ negative	6 (10.3%)/52 (89.7%)	11 (24.4%)/34 (75.6%)	0.1
**HBV.DNA2**, copies/ml ≤ 10^4/>10^4	45 (77.6%)/13 (22.4%)	33 (73.3%)/12 (26.7%)	0.789
**HCVAb**, positive/ negative	4 (6.9%)/54 (93.1%)	0 (0%)/45 (100%)	0.2
**Child-Pugh class**, A/B	46 (79.3%)/12 (20.7%)	40 (88.9%)/5 (11.1%)	0.302
**TBIL**, mmol/l	14.4 [11.8, 20.0]	14.6 [11.8, 19.7]	0.537
**ALB**, g/l	37.0 [33.0, 40.0]	38.0 [36.0, 41.0]	0.095
**ALT**, m/l	44.0 [26.0, 72.8]	35.0 [25.0, 62.0]	0.767
**AST**, m/l	41.5 [29.3, 66.0]	40.0 [27.0, 80.0]	0.489
**PT**, s	13.2 [12.8, 14.0]	13.0 [12.5, 13.4]	**0.038**
**INR**	1.00 [0.950, 1.08]	0.990 [0.940, 1.01]	**0.041**
**InAFP**	4.04 [1.90, 5.32]	4.62 [4.22, 7.23]	**0.004**
**CEA**, ng/ml	2.60 [1.85, 4.08]	2.60 [1.90, 3.90]	0.535
**CA125** U/ml	12.0 [8.85, 17.6]	12.0 [9.30, 23.1]	0.335
**CA19-9**, U/ml	16.9 [9.15, 31.1]	16.4 [6.90, 26.6]	0.517
**Liver cirrhosis**, Absent/ Present	28 (48.3%)/30 (51.7%)	27 (60.0%)/18 (40.0%)	0.325
**Ascites**, Absent/ Present	49 (84.5%)/9 (15.5%)	38 (84.4%)/7 (15.6%)	1
**Tumour number**, Solitary/ Multiple	43 (74.1%)/15 (25.9%)	38 (84.4%)/7 (15.6%)	0.306
**Tumour diameter**, cm	4.20 [2.40, 6.50]	4.90 [2.80, 8.10]	0.102
**Tumour margin**, Non smooth/ Smooth	37 (63.8%)/21 (36.2%)	32 (71.1%)/13 (28.9%)	0.567
**Tumour capsule**, Absent/ Present	29 (50.0%)/29 (50.0%)	27 (60.0%)/18 (40.0%)	0.417
**Hemorrhage**, Absent/ Present	55 (94.8%)/3 (5.2%)	38 (84.4%)/7 (15.6%)	0.153
**Venous invasion**, Absent/ Present	47 (81.0%)/11 (19.0%)	36 (80.0%)/9 (20.0%)	1
**Early enhancement**, Absent/ Present	9 (15.5%)/49 (84.5%)	13 (28.9%)/32 (71.1%)	0.162
**Washout**, Absent/ Present	15 (25.9%)/43 (74.1%)	15 (33.3%)/30 (66.7%)	0.542

**Note**: HbsAg, Hepatitis B surface antigen; HBeAG, hepatitis B e antigen; HCVAb, Hepatitis C Virus Antibody; TBIL, total bilirubin; ALB, albumin; ALT, alanine transarninase; AST, Aspartate transaminase; PT, prothrombin time; INR, international normalized ratio; AFP, alpha-fetoprotein; CEA, carcinoembryonic antigen; CA125, cancer antigen 125; CA19-9, carbohydrate antigen 19 − 9; TYMS, thymidylate synthase

### Diagnostic performance of two models

**Fig. 2:**
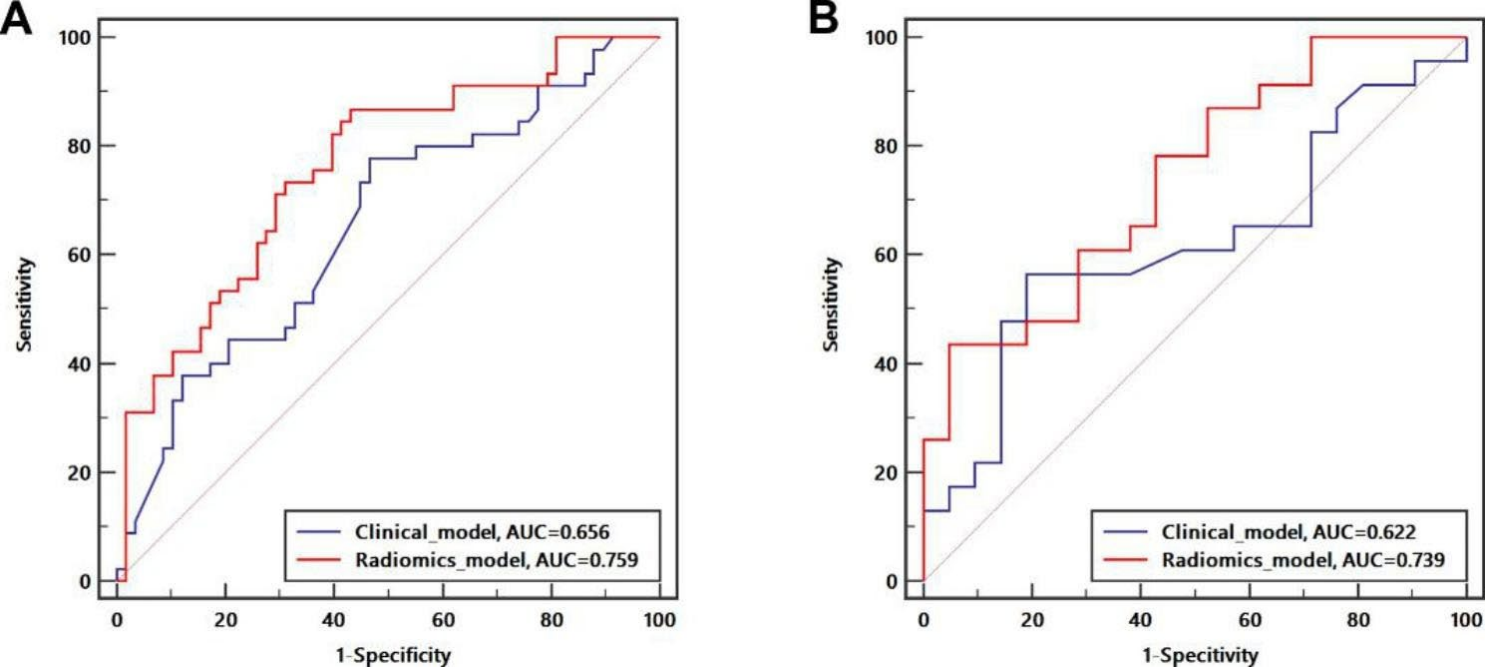
ROC curves comparing the two models in training (**A**) and validation (**B**) set

**Fig. 3:**
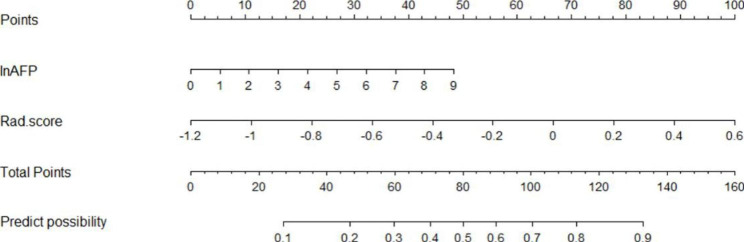
The radiomics nomogram, combining InAFP and Rad score developed in the training set

The results showed that the radiomics nomogram constructed by the Rad score and InAFP generated the highest performance for predicting TYMS status in the training and validation sets. In the training set, the AUCs of the clinical model and radiomics nomogram were 0.656 (95% CI 0.555–0.746) and 0.759 (95% CI 0.665–0.838), respectively. In the validation set, the AUCs of the clinical model and radiomics nomogram were 0.622 (95% CI 0.463–0.764) and 0.739 (95% CI 0.585–0.860), respectively (Figs. 2 and 3). Therefore, the predictive performance of the radiomics nomogram was superior to that of the clinical model in both sets (P < 0.05).

**Fig. 4:**
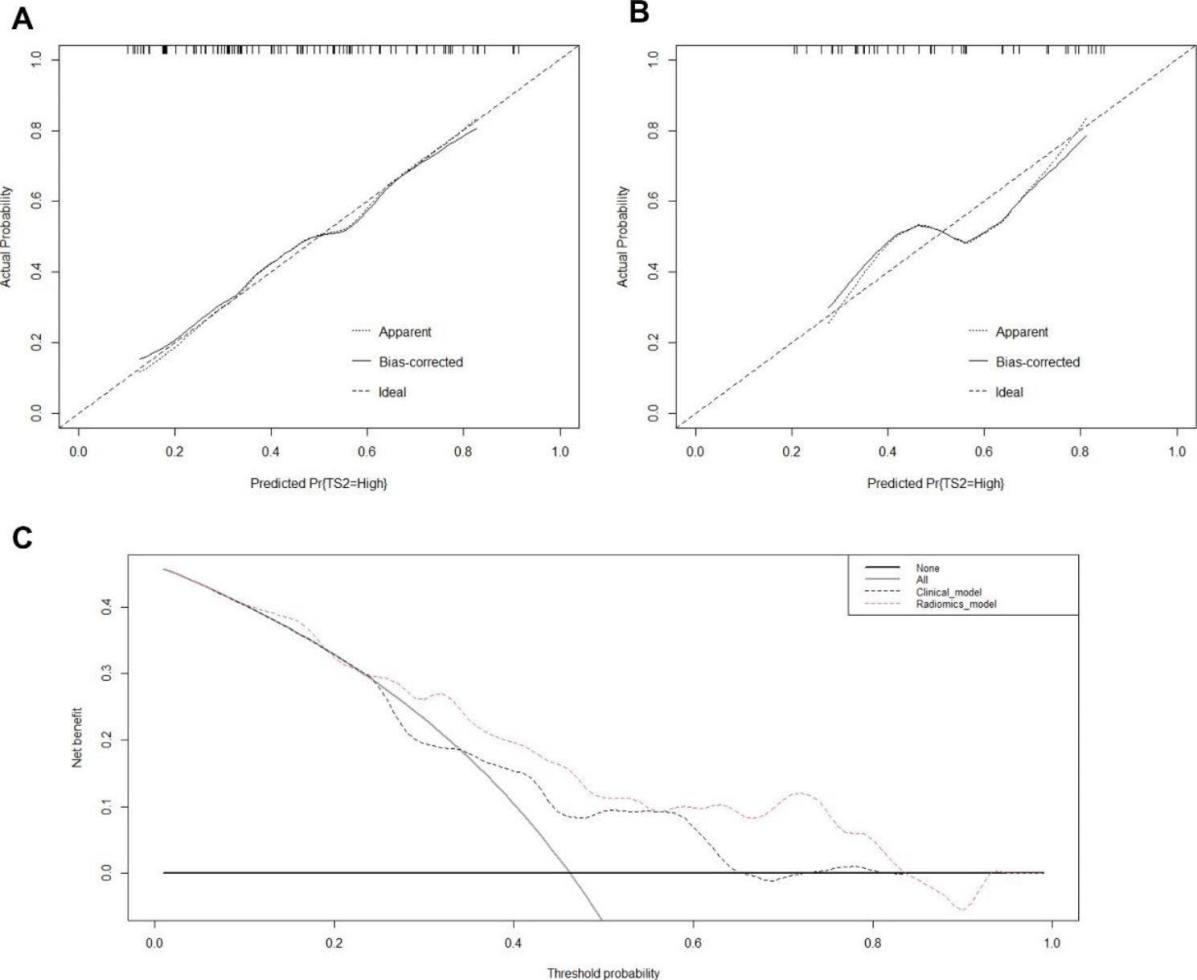
Calibration curves of the nomogram on the training set (**A**) and validation set (**B**). (**C**) Decision curve analysis for the nomogram in the total dataset

The calibration curves showed good consistency between the nomogram estimated by the radiomics model and the actual frequencies of TYMS status in both the training and validation sets. DCA showed that the radiomics nomogram had a higher overall net benefit for predicting TYMS status than the clinical model across the majority of the range of reasonable threshold probabilities (Fig. 4). More details about the predictive performance of the two models are described in detail in Table 3.

**Table 3:** The diagnostic performance of the Clinical model and Radiomics nomogram in training and validation set. **Note**: AUC, the area under the curve; CI, Confidence interval; PLR, positive likelihood ratio; NLR, negative likelihood ratio

		Cut-off	AUC (95% CI)	Sensitivity (95% CI)	Specificity (95% CI)	PLR (95% CI)	NLR (95% CI)
**Training set**	Clinical model	0.41	0.656 (0.555–0.746)	77.78 (62.9–88.8)	53.45 (39.9–66.7)	1.67 (1.22–2.29)	0.42 (0.23–0.76)
	Radiomics nomogram	0.32	0.759 (0.665–0.838)	86.67 (73.2–94.9)	56.9 (43.2–69.8)	2.01 (1.46–2.76)	0.23 (0.11–0.51)
**Validation set**	Clinical model	0.53	0.622 (0.463–0.764)	56.52 (34.5–76.8)	80.95 (58.1–94.6)	2.97 (1.15–7.69)	0.54 (0.32–0.89)
	Radiomics nomogram	0.68	0.739 (0.585–0.860)	43.48 (23.2–65.5)	95.24 (76.2–99.9)	9.13 (1.27–65.39)	0.59 (0.41–0.86)

## Discussion

The present study showed that the Gd-DTPA contrast-enhanced MR-based radiomics model, which incorporates the Rad score and InAFP, has a favourable predictive value for predicting TYMS status in HCC with AUCs of 0.759 and 0.739 in the training and validation sets, respectively. In comparison to the clinical model, the radiomics model showed overall superiority in the evaluation of AUC in both the training and validation sets. The calibration curves showed good consistency between the nomogram estimated by the radiomics model and the actual frequencies of the TYMS status in both the training and validation sets. Decision curve analysis showed that the radiomics nomogram outperformed the clinical model in terms of clinical usefulness. To the best of our knowledge, this is the first report that uses radiomics to establish a nomogram for predicting TYMS status in HCC.

Numerous studies have shown that models combining textural or radiomics features with clinical factors are more comprehensive and reliable than clinical models [17–19]. The reason why radiomics models have better predictive performance is not clear. However, most studies have supposed that this is directly related to the hypothesis of radiomics [17–19]. The underlying hypothesis of radiomics is that genomic and proteomic patterns can be expressed in terms of macroscopic image-based features. We can infer phenotypes or gene–protein signatures, possibly containing prognostic information, from the quantitative analysis of medical image data. Our conclusion confirms this hypothesis. On the basis of the clinical model, the dimension of radiomics was added, which greatly improved the performance of predicting TYMS.

Our results show that InAFP was the only independent predictor of TYMS status, and InAFP was significantly increased in the high TYMS group. The results indicated that AFP has a strong correlation with drug resistance to fluorouracil, and AFP may be an important marker for patient selection of treatment regimens containing fluorouracil. AFP is an important biomarker for the diagnosis and prognosis of HCC. AFP also plays an important role in inhibiting the immune response in vivo, and these effects lead to MDR in HCC cells [20]. Moreover, AFP can be used as a biomarker for HCC drug therapy. A phase II clinical trial was conducted to evaluate the efficacy of intravenous fluorouracil in the treatment of HCC; i.v. fluorouracil was well tolerated and induced a durable partial response in 31% (5 of 16) of patients with HCC who had low levels of serum AFP; and the treatment regimen was ineffective in patients with HCC who had high levels of serum AFP [21]. Yan Wang et al. [22] evaluated the prognostic significance of AFP status in HCC patients after transarterial chemoembolization (TACE) and receiving the chemotherapeutic agents of TACE, including fluorouracil and cisplatin, and reported that patients with AFP-negative status have a better treatment response and prognosis after TACE than AFP-positive HCC patients. Takahiro Yamasaki et al. reported the prognostic factors in patients with advanced HCC receiving hepatic arterial infusion chemotherapy (HAIC) using low-dose cisplatin (CDDP) and 5-fluorouracil (5 - FU) with/without leucovorin (or isovorin), indicated that AFP level was a significant prognostic factor, and suggested that patients with a lower AFP level were suitable candidates for HAIC [23].

Our study has certain guiding significance for clinicians to make treatment decisions. HCC is mostly diagnosed at advanced stages of the disease, and chemotherapeutic drugs are one of the major therapeutic options for the treatment of patients with advanced HCC. Fluorouracil has been verified to prolong the survival of these patients [24]. Meanwhile, several preclinical studies have shown that TYMS expression levels are a key determinant for therapeutic responsiveness to fluorouracil [8–10]. Moreover, it is difficult to determine the status of TYMS because pathological biopsy is usually not recommended for advanced HCC. Therefore, our noninvasive and simple model can be a potential alternative tool.

Our study had several limitations. First, all included patients had surgically resected HCC, and the predictive performance of the model in unresectable HCC still needs to be further validated. Second, the number of samples was still limited compared to the large number of features. A large-scale clinical study enrolling more samples would help validate and improve the applicability of our model as an effective tool to predict TYMS status. Finally, sample selection bias was unavoidable in this retrospective study. Therefore, a prospective study should be conducted to further prove the practicability of the model.

In conclusion, the MR-based radiomics nomogram, a noninvasive prediction tool that incorporates the Rad score and InAFP, shows favourable predictive efficacy for TYMS status in HCC, which might be helpful for the selection of chemotherapy drugs and the prediction of prognosis.

## Data Availability

The datasets used and/or analysed during the current study are available from the corresponding author on reasonable request.

## Abbreviations and acronyms

TYMS: Thymidylate synthase
HCC: Hepatocellular carcinoma
MRI: Magnetic resonance imaging
MDR: Multidrug resistant
TopoII: DNA topoisomerases II
βIII-tubulin: Tubulin beta-III
P-gp: P-glycoprotein
dUMP: Deoxyuridine monophosphate
GLDM: Gray-level dependence matrix
GLCM: Gray-level cooccurence matrix
GLRLM: Gray-level run length matrix
GLSZM: Gray-level size zone matrix
NGTDM: Neigbouring gray tone difference matrix
ICC: Intraclass correlation coefficient
LASSO: The least absolute shrinkage and selection operator algorithm
AFP: Alpha-fetoprotein
HbsAg: Hepatitis B surface antigen
ROC: Receiver operating characteristic
AUC: The area under the curve
CI: Confidence interval
DCA: Decision curve analysis
VOIs: Volume of interest
